# Spatial deciphering of the transcriptomic heterogeneity of tumor spread through air spaces in lung cancer

**DOI:** 10.3389/fphar.2025.1567527

**Published:** 2025-07-25

**Authors:** Wenhao Wang, Wenhao Zhou, Jingli Fan, Tao Jiang, Guang Yang, Congcong Song, Siwei Xu, Haitao Luo, Huining Liu

**Affiliations:** ^1^ Department of Thoracic Surgery, The First Hospital of Hebei Medical University, Shijiazhuang, Hebei, China; ^2^ Shenzhen Engineering Center for Translational Medicine of Precision Cancer Immunodiagnosis and Therapy, YuceBio Technology Co., Ltd, Shenzhen, China

**Keywords:** spread through air spaces, non-small-cell lung cancer, digital spatial profiling, spatial heterogeneity, spatial transcriptome

## Abstract

**Background:**

Spread through air spaces (STAS) represents a novel invasion mechanism in adenocarcinoma that considerably influences lung cancer clinical outcomes; however, studies of its mechanisms at the spatial level are lacking.

**Methods:**

We used the NanoString GeoMx digital spatial profiling (DSP) technology to conduct a spatial transcriptomic analysis of surgically resected tissues from non-small-cell lung cancer (NSCLC) patients with or without STAS.

**Results:**

Compared with tumor nests in non-STAS patients, *HLA-DRB5* and *RASGRF1* were significantly less expressed in compartments of STAS, suggesting their inhibitory roles in the occurrence of STAS. Meanwhile, an increase in CD4 T memory cells and a decrease in B cells were observed in the tumor immune microenvironment of STAS. Furthermore, distinct molecular profiles were observed between tumor cells in tumor nests and in air spaces in STAS patients, which was highlighted by the elevated *ITGA2* expression in the air spaces. These results were validated in an independent cohort by multiplex immunofluorescence stainings.

**Conclusion:**

This study is the first to use DSP to analyze spatial transcriptomic profiles of NSCLC tumor nests and air space tumors, and it identifies potential module features that may be used for STAS identification and prognosis.

## Background

Over the last decade, we have made significant progress in our understanding and research of lung cancer biology, leading to noteworthy improvements in patient outcomes. However, lung cancer continues to be the principal cause of cancer-related mortality. In 2023, the United States was projected to have 238,340 new cases of lung cancer and 127,070 lung-related deaths (Si et al., 2023). Among patients undergoing surgical removal, those with early-stage lung cancer often face less-than-ideal prognoses. Clinical thoracic surgeons have become increasingly focused on the phenomenon of spread through air spaces (STAS), defined as tumor cells—micropapillary structures, solid nests, or single cells—spreading within air spaces in the lung parenchyma beyond the edge of the main tumor (Kadota et al., 2015). Thus, it has been recognized as a fourth mode of invasive lung adenocarcinoma (Travis et al., 2015), prompting a reevaluation of traditional TNM tumor staging.

Numerous retrospective studies have verified that STAS significantly impacts the recurrence-free survival (RFS) and overall survival (OS) of patients with non-small-cell lung cancer (NSCLC), with STAS-positive patients experiencing a shorter prognosis (Wang et al., 2019; Terada et al., 2019; Toyokawa et al., 2018). Recently, a study identified STAS as an independent risk factor for postoperative recurrence, noting that lobectomy, compared to limited resection, offers STAS-positive patients better RFS and lower cumulative mortality rates, effectively reducing local recurrence (Toyokawa et al., 2018; Eguchi et al., 2019). Thus, the presence of STAS not only influences patient prognosis but also surgical treatment decisions (Travis et al., 2015). However, the covert nature of STAS presents a challenge in accurately identifying it through clinical examinations or external tumor sample analysis. This underscores the urgent need of an in-depth study into its biomarkers and molecular mechanisms.

The mechanisms underlying early-stage lung cancer in STAS patients are frequently associated with *ALK* and *ROS1* rearrangements and *KRAS* mutations (Kadota et al., 2019; Wang S. et al., 2022). Certain studies have pinpointed the expression of the *CXCL8* and *CPB2* genes as prognostic markers for STAS, with hypoxia Von Hippel–Lindau (VHL) targets, the protein kinase C (PKC) pathway, and pyrimidine metabolism pathways identified as characteristic signaling pathways (Lee et al., 2018; Zeng et al., 2022). STAS-positivity correlates significantly with the protein-level expression of the metastasis-associated 1 protein (MTA1) in lung adenocarcinoma (Liu et al., 2018), and high MMP-7 protein expression has been recognized as an independent predictor of STAS occurrence (Yamada et al., 2023). However, large-scale current studies have been conducted on the molecular mechanisms of STAS without adequately exploring the spatial molecular differences among different cell populations in STAS and non-STAS (NSTAS) patients, nor the distinctions between airspace (AS) and tumor nests (TNs). Consequently, further investigation into the mechanisms and predictive biomarkers of lung cancer STAS at the spatial level is crucial.

In this research, we utilized the NanoString GeoMx digital spatial profiling (DSP) spatial transcriptomics technology to delineate the transcriptomic feature profiling of TNs in STAS and NSTAS patients and uncover the transcriptomic characteristics of the AS and TNs in STAS patients, thus interpreting, for the first time, the molecular mechanisms and biomarkers of STAS occurrence in a spatial dimension.

## Materials and methods

### Patient information

Treatment-naive NSCLC patients who underwent thoracoscopic minimally invasive surgery at the Department of Thoracic Surgery of the First Hospital of Hebei Medical University from 2021 to 2023 were enrolled. Six patients were found to have STAS during pathological examination, and six patients did not have STAS during the same surgical period. All samples were formalin-fixed paraffin-embedded (FFPE) and then consecutively sliced, with a thickness of 4–6 μm per slice. Hematoxylin and eosin (HE) staining was performed on the paraffin-embedded tissue sections to ensure that the samples contained TNs, normal tissues (N), and AS regions. For spatial transcriptomic analysis, we employed the NanoString GeoMx Digital Spatial Profiling (DSP) technology. Written informed consent was obtained from all participants.

### Digital spatial profiling

Sections marked by HE staining were selected for DSP analysis with the GeoMx Cancer Transcriptome Atlas (CTA, Version 2.0), which encompasses a panel of 1,833 genes. This CTA panel was applied to the slides and further processed using the GeoMx Solid Tumor TME Morphology Kit (NanoString, United States, Catalog # GMX-RNA-MORPH-HST-12) to distinguish between distinct cell morphologies. Epithelial cells were identified by PanCK-positive staining, immune cells were identified by CD45-positive staining, and cell nuclei were identified by SYTO13-positive staining. Normal and malignant epithelial cells, TNs, and AS were identified by two pathologists. STAS was defined as consisting of micropapillary clusters, solid nests, or single cells beyond the edge of the tumor into air spaces in the surrounding lung parenchyma (Kadota et al., 2015; Travis et al., 2015). Regions of interest (ROIs) were selected and assessed by two pathologists. The PanCK+ and CD45^+^ compartments were segmented with ultraviolet light as the minimum area of illumination or area of interest (AOI). Index oligonucleotides released from each AOI were collected into a 96-well plate for quantification. Libraries were prepared according to NanoString’s instructions and sequenced on a DNBSEQ-T7 platform. AOIs containing over 100 nuclei were filtered. In total, 89 AOIs were analyzed, with 50 AOIs from six STAS patients, which included 16 AOIs from TN malignant epithelial cells (PanCK+ compartments), two AOIs from TN immune cells (CD45^+^ compartments), 18 AOIs from normal epithelial cells (PanCK+ compartments), and 14 AOIs from AS malignant epithelial cells (PanCK+ compartments); 39 AOIs from six NSTAS patients, which included 18 AOIs from TN malignant epithelial cells (PanCK+ compartments), three AOIs from TN immune cells (CD45^+^ compartments), and 18 AOIs from normal epithelial cells (PanCK+ compartments).

### Spatial transcriptomic data analysis

The GeoMx NGS pipeline software V2.0.0.16 was used to convert the sorted FASTQ files into DCC files. The DCC file was then uploaded to the GeoMx DSP system. Analysis was performed on the GeoMx DSP Control Center using the Data Analysis module V.2.4.0.421. The QC of the transcriptomic data included technical signals, technical background, probes, and normalization. When the alignment rate between the reading and the template sequence was less than 80%, we used the technical signal QC to remove AOI. The technical background included three indicators: template-free control (TFC) count, negative probe count, and AOI parameters. TFC counting was used to detect template contamination during library construction. AOIs with a TFC greater than 1,000 were removed. In the CTA experiment, negative probe counting was used to measure the overall technical signal level. The threshold for negative probe counting was four counts. We used 75th percentile normalization to adjust the size of the different AOIs to avoid differences between them. Hierarchical clustering and a correlation matrix were performed using the “ComplexHeatmap” software package (version 2.16.0). Principal component analysis (PCA) was performed using “prcomp of stats” version 4.1.0. For differential expression analysis, edgeR (version 3.42.4) (Robinson et al., 2010) was used, with a threshold |logFC| greater than 0.5 and *p*-value <0.01 to screen for differentially expressed genes (DEGs). Other related graphs were generated by the “ggplot2” software package (version 3.4.2). For functional and pathway annotation and enrichment analysis, DEGs were processed using the clusterProfiler software package (version 4.8.3). The immune cell signature scores were calculated using the GSEA algorithm (version 1.54.0) and deconvoluted by SpatialDecon (version 1.2.0) to estimate the proportion of immune cells in the immune microenvironment.

### Multiplex immunofluorescence (mIF) staining

Samples from 12 independent cohorts were subjected to mIF staining. The 5-μm-thick slides cut from the FFPE blocks were dewaxed with xylene. Then, the slides were rehydrated with a decreasing ethanol series. After rehydration, the slides were fixed with 10% neutral buffered formalin for 10 min. Next, the slides were stained with markers of PanCK, CD20, CD4, HLA-DRB5, N5TE, and ITGA2 (Akoya Biosciences, United States), followed by incubation with blocking proteins for 10 min. After blocking, the slides were incubated with horseradish peroxidase (HRP)-conjugated secondary antibody and tyramide signal amplification (TSA). Finally, the slides were stained with 4′-6′-diamidino-2-phenylindole (DAPI) for 10 min, and images were acquired with Vectra Polaris (Akoya Biosciences, United States).

### Statistical analysis

Dimension reduction analysis was performed using uniform manifold approximation and projection (UMAP) (version 0.2.8.0). The Wilcoxon test was used for comparison of intergroup differences. Correlation analysis was conducted using the Pearson correlation analysis. The significance of OS and progression-free survival (PFS) was analyzed using Kaplan–Meier (K–M) curve analysis based on the TCGA dataset with web tools of GEPIA2 (Tang et al., 2019). The K–M curve was compared using the log-rank test. The median follow-up time was calculated using the reverse K–M method. Statistical significance was set at a *p*-value of <0.05.

## Results

### Spatial transcriptomic detection of STAS and NSTAS

**FIGURE 1:**
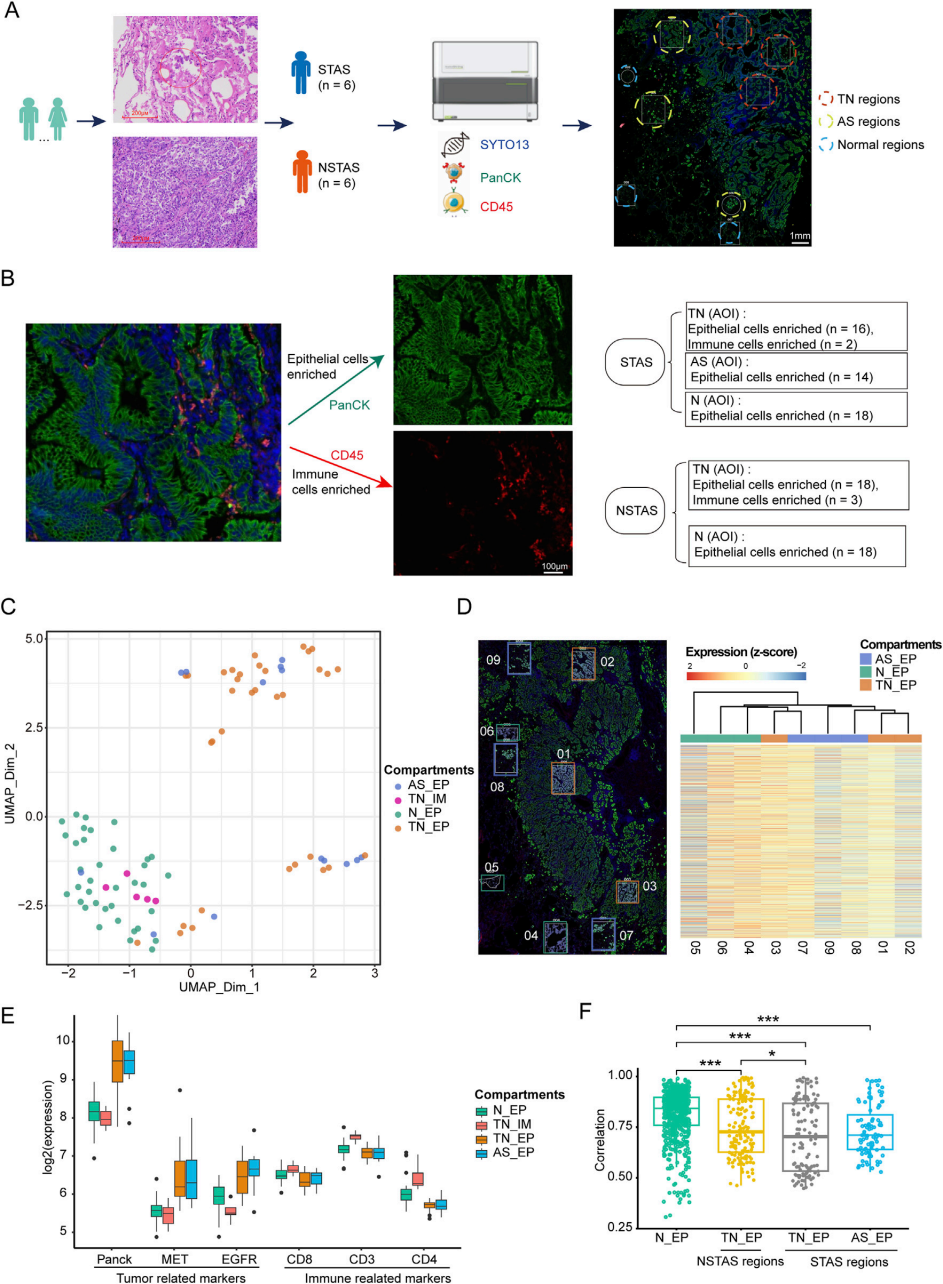
Digital spatial profiling omics detection of STAS and NSTAS. **(A)** Classification of STAS and NSTAS samples, along with the distinction of different regions. **(B)** Segmentation and AOI selection in samples using the DSP technology. **(C)** UMAP of dimensionality reduction analysis for AOI across regions. **(D)** Comparison between the spatial distance of different AOIs in the P1 sample and the molecular clustering results from the transcriptome image. **(E)** Expression of characteristic genes in different regions. **(F)** Correlation analysis of Panck+ AOIs in N_EP, TN_EP, and AS_EP. Samples from normal tissue are marked in green, TN_EP samples from NSTAS are marked in yellow, TN_EP samples from STAS are marked in gray, and AS_EP samples from STAS are marked in blue. STAS, spread through air spaces; NSTAS, non-spread through air spaces; AOI, area of interest; UMAP, uniform manifold approximation and projection; TN_EP, tumor nest malignant epithelial cell-enriched compartments; AS_EP, airspace malignant epithelial cell-enriched compartments; N_EP, normal epithelial cell-enriched compartments; TN_IM, tumor nest immune cell-enriched compartments.

This study included a total of 12 NSCLC patients, divided equally into six cases of STAS and six cases of NSTAS. Both groups maintained a balanced gender ratio and had an average age of 61 years (Table 1; Supplementary Table S1). The pathological TNM staging for these patients ranged from stages I to II. Based on the results of HE staining and mIF, the samples were classified into TN regions, AS regions, and normal regions, which were confirmed by two pathologists (Figure 1A). Using mIF staining with PanCK and CD45 antibodies, we performed spatial segmentation of the tumor and immune regions, followed by spatial transcriptomic analysis. DSP was then utilized to analyze the spatial transcriptome of 1,833 genes, with a total of 89 AOIs selected for analysis (Figure 1B). TN malignant epithelial cell-enriched compartments were denoted as TN_EP, AS malignant epithelial cell-enriched compartments were denoted as AS_EP, normal epithelial cell-enriched compartments were denoted as N_EP, and immune cell-enriched compartments within the TN were labeled TN_IM. UMAP analysis distinctly differentiated malignant epithelial cells from normal cell clusters, highlighting the unique molecular features in malignant cells compared to those in normal epithelial or immune cells (Figure 1C).

**TABLE 1:** Summary of clinical characteristics of patients.

Characteristics	Overall (*n* = 12)
Median age (range), years	61 (37–78)
Sex, *n* (%)	
Male patients	6 (50%)
Female patients	6 (50%)
STAS status, *n* (%)	
STAS	6 (50%)
NSTAS	6 (50%)
TNM stage, *n* (%)	
T1aN0M0	3 (25.0%)
T1bN0M0	8 (66.7%)
T1bN1M0	1 (8.3%)
LNm stage, *n* (%)	
Yes	1 (8.3%)
No	11 (91.7%)

STAS, spread through air spaces; TNM, tumor node metastasis; LNm, lymph node metastasis.

To deeply explore intra-tumor heterogeneity (ITH) at the spatial level, we mapped the molecular profiles of spatial distribution areas in each sample (Figure 1D). Spatial clustering analysis revealed that the AS_EP compartments shared a more similar expression pattern with the adjacent TN_EP, aligning with our hypothesis that AS_EP may progress from TN_EP. When compared to N_EP and TN_IM, the TN_EP and AS_EP showed greater enrichment of malignant genes, such as *Panck*, *MET*, and *EGFR*, and a reduced expression of immune-related genes (Figure 1E). Intra-group correlation analysis indicated that both TN_EP and AS_EP exhibited significantly higher heterogeneity than N_EP, with TN_EP showing greater heterogeneity in STAS than in NSTAS (Figure 1F).

### The T_EP group exhibits greater cellular dysfunction and more metabolic abnormalities than the N_EP group in NSCLC

**FIGURE 2:**
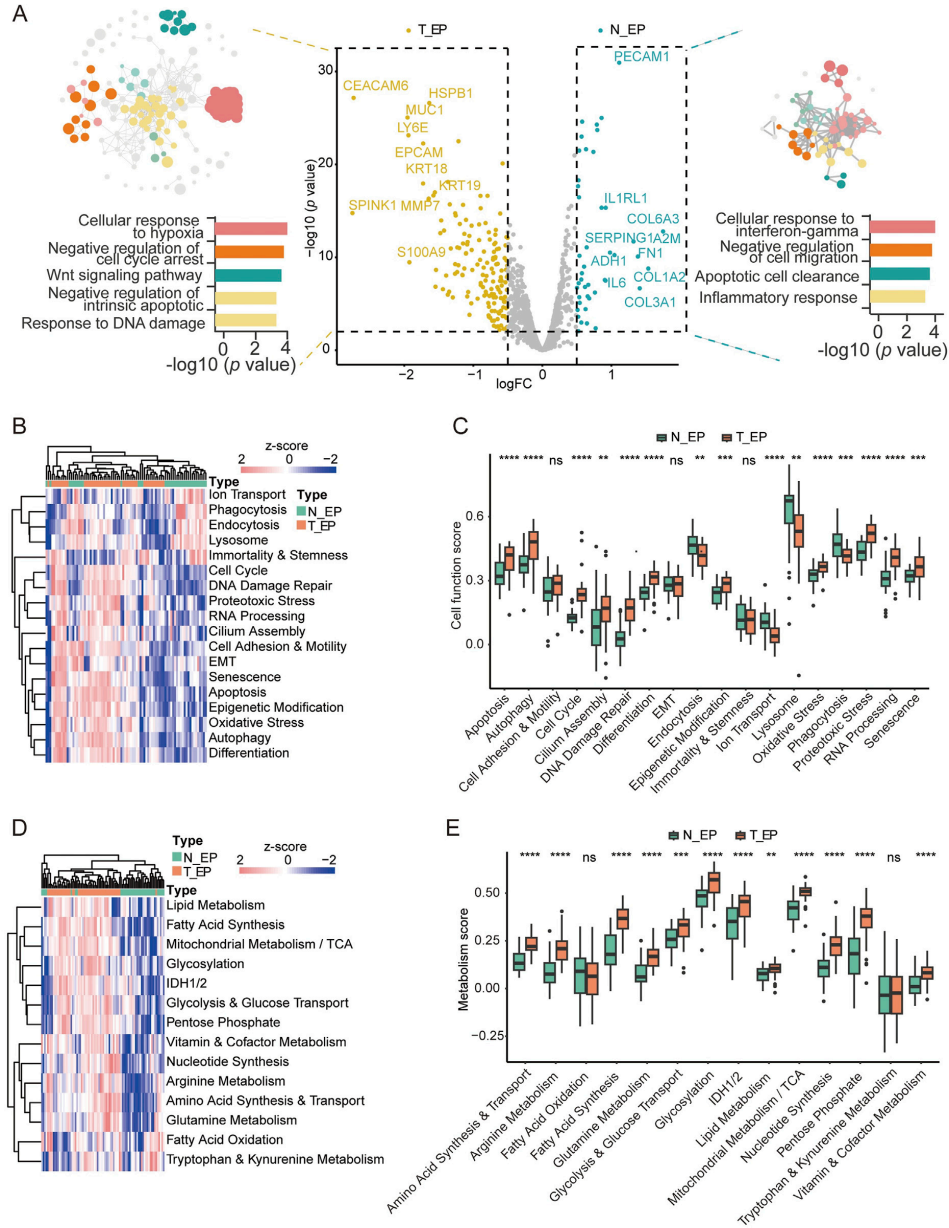
Digital spatial profiling analysis of T_EP and matched N_EP in patients with NSCLC. **(A)** DEGs and the corresponding enrichment pathway analysis between the T_EP and N_EP groups. **(B)** Heatmap of the cell function signature between the T_EP and N_EP groups. **(C)** Boxplot representing the signature comparison regarding the cell function between the T_EP and N_EP groups. **(D)** Heatmap of the metabolic signature regarding comparison between the T_EP and N_EP groups. **(E)** Boxplot representing the comparison of the metabolism-related signature between the T_EP and N_EP groups. T_EP, tumor epithelial region including TN_EP and AS_EP, tumor nest malignant epithelial cell-enriched compartments; AS_EP, airspace malignant epithelial cell-enriched compartments; N_EP, normal epithelial cell-enriched compartments; DEGs, differentially expressed genes; ns, *p* > 0.05; **p < 0.01; ***p < 0.001; ****p < 0.0001.

To explore the characteristics of NSCLC occurrence, we compared the T_EP (both the TN_EP and the AS_EP) and the N_EP compartments. In T_EP, there was significant upregulation of genes associated with malignancy, such as *EPCAM*, *HSPB1*, *CEACAM6*, *MUC1*, and *LY6E* (Rehulkova et al., 2023; Huang et al., 2018; Wang et al., 2023; Jing et al., 2019; Yeom et al., 2016), implicating their roles in tumor initiation (Figure 2A; Supplementary Table S2). Enrichment analysis indicated promising enrichment of tumor cells in pathways including the cellular response to hypoxia, negative regulation of cell cycle arrest, Wnt signaling, negative regulation of intrinsic apoptosis, and response to DNA damage. In contrast, T_EP compartments were found to be lacking in the pathways related to the cellular response to interferon-gamma, negative regulation of cell migration, apoptotic cell clearance, and the inflammatory response.

To further validate our results, we conducted a cellular signature analysis. Cellular functional pathways. such as apoptosis, cell cycle regulation, DNA damage repair, differentiation, and oxidative stress were significantly observed in malignant cells (Figures 2B,C; Supplementary Table S3), and this was consistent with the trend observed in our pathway enrichment results. Furthermore, many metabolic pathways such as amino acid synthesis and transport, arginine metabolism, glycolysis and glucose transport, glutamine metabolism, and fatty acid synthesis were significantly observed in malignant cells (Figures 2D,E; Supplementary Table S4). These results may indicate that cellular dysfunction and metabolic dysregulation are implicated in epithelial cell carcinogenesis.

### The expression of *HLA-DRB5* was associated with the absence of STAS

**FIGURE 3:**
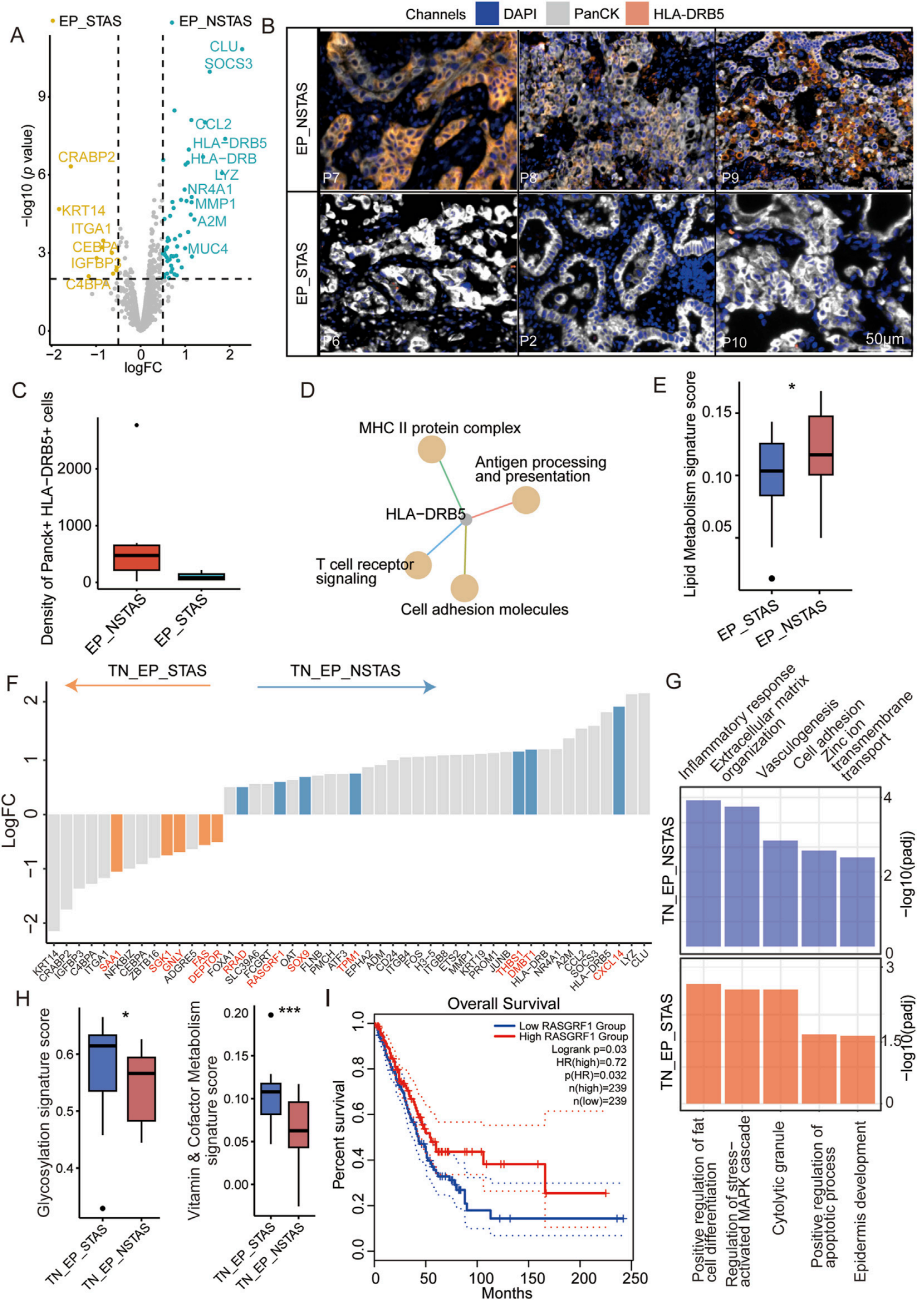
Molecular characteristic comparison in the TN_EP compartments between STAS and NSTAS. **(A)** Volcano diagram representing the DEGs of malignant EP compartments between STAS and NSTAS. **(B)** Representative images of EP_NSTAS and EP_STAS tissues showing DAPI (blue), PanCK (white), and HLA-DRB5 (orange) staining, as determined by mIF. Scale bar, 50 μm. **(C)** Density of the Panck- and HLA-DRB5-positive cells among groups using mIF data. **(D)** Pathway network that involves *HLA-DRB5*. **(E)** Boxplot of the lipid metabolism signature score between STAS and NSTAS. **(F)** DEGs between TN_EP_STAS and TN_EP_NSTAS. The unique DEGs within the TN_EP regions are highlighted in red. **(G)** Functional pathway enrichment of TN_EP_STAS and TN_EP_NSTAS. **(H)** Boxplot of the glycolysis and vitamin and cofactor metabolism signature between TN_EP_STAS and TN_EP_NSTAS. **(I)** Survival curves of OS in LUAD with *RASGRF1* expression. DEGs, differentially expressed genes; EP_STAS, malignant epithelial cell-enriched compartments of STAS; EP_NSTAS, malignant epithelial cell-enriched compartments of NSTAS; TN_EP_STAS, tumor nest malignant epithelial cell-enriched compartments of STAS; TN_EP_NSTAS, tumor nest malignant epithelial cell-enriched compartments of NSTAS; OS, overall survival; DFS, disease-free survival; LUAD, lung adenocarcinoma. *p < 0.05; ***p < 0.001.

Given the clinical phenomena of STAS and NSTAS in NSCLC, we explored the molecular characteristics within the malignant epithelial regions. Comparing epithelial cell-enriched compartments from the malignant EP_STAS group (containing the TN_EP_STAS and AS_EP_STAS subgroups) and the malignant EP_NSTAS group (containing the TN_EP_NSTAS subgroup), we identified 66 DEGs between STAS and NSTAS (Figure 3A; Supplementary Table S5). *CRABP2*, *KRT14*, and *CEBPA* expressions were significantly increased in STAS tumor cells. Conversely, *CLU*, *SOCS3*, and genes from the HLA-DRB family were significantly highly expressed in NSTAS tumor cells. To further validate these findings, we assessed the expression levels of the HLA-DRB5 protein in the tissue using mIF. The results showed that HLA-DRB5 expression was similarly upregulated in epithelial cells within the EP_NSTAS compartment compared to those in the EP_STAS compartment (Figures 3B, C). Survival analysis using the TCGA database showed that patients with a high expression of *HLA-DRB5* had significantly prolonged disease-free survival (DFS) (*p* = 0.0012) and OS (*p* = 0.022) (Supplementary Figure S1), which is consistent with the poorer prognosis of STAS. The involvement of *HLA-DRB5* in pathways, including the formation of the MHC II protein complex and antigen processing and presentation, suggests a more severely suppressed immune response in STAS (Figure 3C), hinting that the expression of the *HLA-DRB5* gene in epithelial cells may guide adaptive immunity through antigen presentation and regulate adaptive immunity through PD-L1 (Shenoy et al., 2021). Metabolically, STAS patients exhibit significantly lower lipid metabolism than NSTAS patients (Figure 3D; Supplementary Table S7).

As is well-known, accurate comparison of the TN_EP regions between NSTAS and STAS is challenging with bulk RNA and single-cell RNA sequencing. Using DSP for spatial selection, we excluded the AS_EP-related region and focused solely on comparing the TN_EP compartment between STAS and NSTAS. A total of 78 DEGs were identified between TN_EP_STAS and TN_EP_NSTAS. Compared with different genes in EP_STAS and EP_NSTAS, five unique genes showed high expression in the TN_EP_STAS compartment (e.g., SAA1, SGK1, and DEPTOR) and seven unique genes showed high expression in the TN_EP_NSTAS compartment (e.g., RASGF1, THBS1, and RRAD) (Figure 3E), which demonstrates the necessity of using spatial omics to explain the differences in TN between STAS and NSTAS. Enrichment analysis of these DEGs revealed that the cell adhesion, vasculogenesis, and inflammatory response pathways were significantly enriched in the TN_EP_NSTAS compartment, whereas the regulation of cell apoptosis, epidermis development, and MAPK cascade activation pathways were significantly enriched in the TN_EP_STAS compartment (Figure 3F). Additionally, metabolic pathway analysis revealed that glycosylation and vitamin metabolism pathways were increased in the TN_EP_STAS compartment (Figure 3G; Supplementary Table S8). Finally, using the TCGA clinical cohort, we identified that high expression of *RASGRF1* significantly prolongs the OS of lung adenocarcinoma (LUAD) patients (Figure 3H); *RASGRF1* is a gene involved in the inflammatory response. In summary, we propose that the expression of *HLA-DRB5* in malignant EP compartments and *RASGRF1* in the TN_EP compartments may be associated with the occurrence of STAS and reveal the different tumor epithelial cell-enriched compartments’ characteristics of the NSTAS and STAS groups.

### Molecular changes in the progression from TN_EP to AS_EP in STAS

**FIGURE 4:**
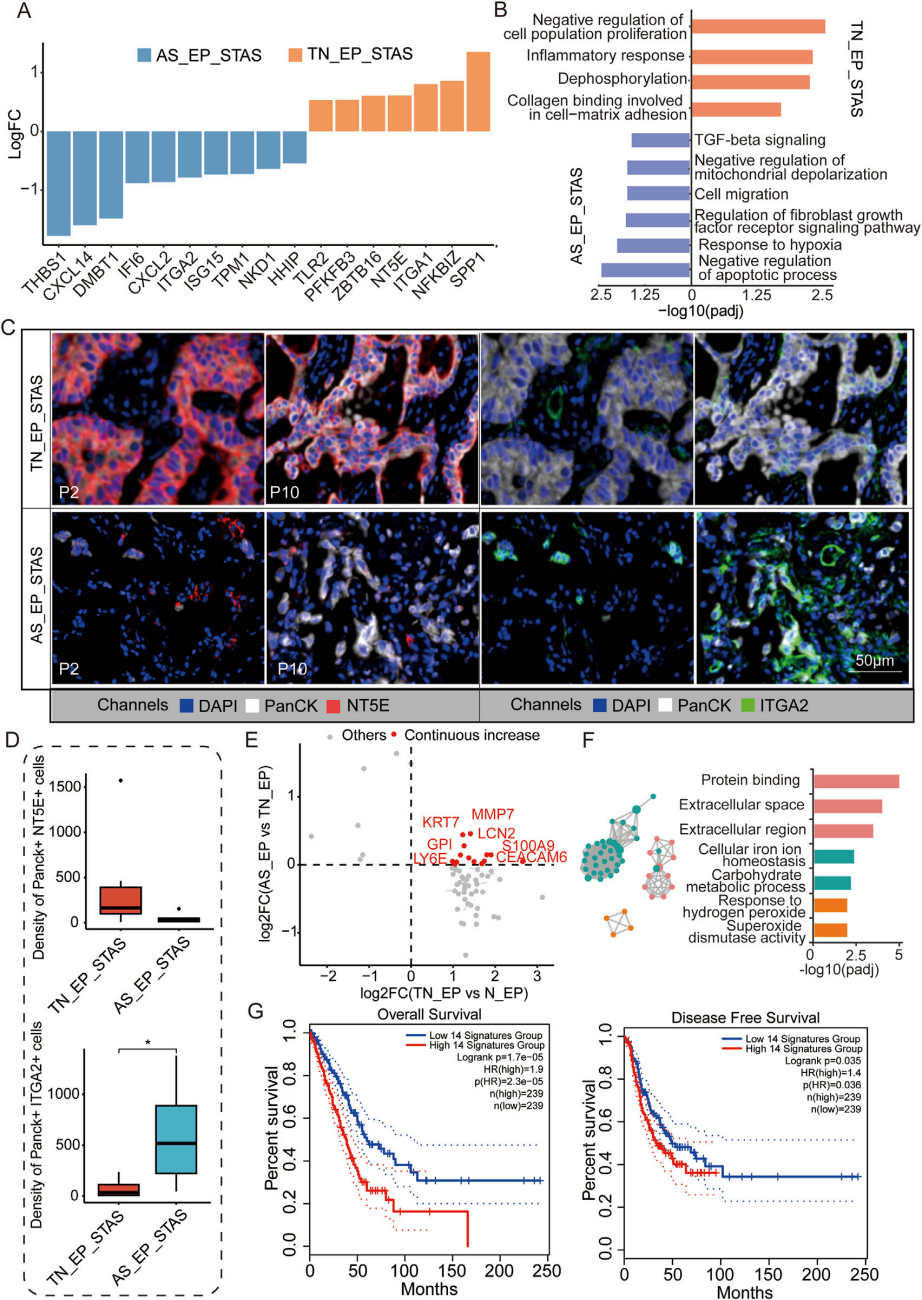
Molecular characteristic comparison between TN_EP_STAS and AS_EP_STAS. **(A)** Hist. diagram displays the DEGs between AS_EP and TN_EP in the STAS group. **(B)** Functional pathway enrichment based on DEGs between AS_EP and TN_EP in the STAS group. **(C)** Representative images from P2 and P10 for DAPI (blue), PanCK (white), NT5E (red), and ITGA2 (green) staining, as determined by mIF. Scale bar, 50 μm. **(D)** Density of the PanCK- and NT5E-positive and Panck- and ITGA2-positive cells among groups using mIF data. **(E)** A quadrant diagram illustrating the DEGs displaying four distinct patterns of change from normal tissue to AS. Genes showing sustained and significant upregulation were defined as those with a continuous increase in gene expression and are highlighted in red. **(F)** Functional pathway enrichment based on the abovementioned genes during STAS progression. **(G)** Impact of a continuously upregulated gene signature during AS_EP progression on OS and DFS in NSCLC patients. DEGs, differentially expressed genes; TN_EP_STAS, tumor nest malignant epithelial cell-enriched compartments of STAS; AS_EP_STAS, airspace malignant epithelial cell-enriched compartments of AS. *p < 0.05; ***p < 0.001; ****p < 0.0001.

To further explore the mechanisms of airspace dissemination in STAS patients, we analyzed DEGs between the TN_EP and AS_EP compartments in STAS patients (Figure 4A). Some of the genes, such as *THBS1*, *CXCL14*, and *DMBT1*, were notably enriched in AS_EP_STAS, suggesting their crucial role in the process of tumor cell spread into air spaces. Pathway enrichment analysis revealed that the negative regulation of apoptosis, the response to hypoxia, cell migration, and the TGF-β signaling pathway were significantly enriched in AS_EP_STAS, indicating that these pathways are involved in AS dissemination (Figure 4B). In contrast, AS_EP_STAS showed a lack of significant enrichment in pathways associated with the negative regulation of proliferation, the inflammatory response, or collagen binding involved in cell–matrix adhesion. To further assess the robustness of our results, *NT5E* and *ITGA2* were identified as specifically upregulated genes in the AS_EP_STAS and TN_EP_STAS compartments, respectively (Supplementary Figure S2), and they were validated using mIF. Consistent with our expectations, NT5E exhibited an upward trend in the epithelial compartment of TN_EP_STAS, whereas ITGA2 was markedly upregulated in the AS_EP_STAS compartment (Figures 4C,D; Supplementary Table S9).

Signature score analysis indicated a higher EMT signature score in the AS_EP_STAS compartment, implying that the EMT program is activated in subsets of primary tumor cancer cells (Supplementary Figure S3; Supplementary Table S10). EMT activation leads to the loss of their characteristic apical–basal polarity, cell–cell junctions, and adhesion to the basement membrane, thus acquiring traits that enable migration and invasion (Mittal, 2018). Moreover, fatty acid synthesis and TCA cycle signatures were significantly reduced in the AS_EP_STAS compartment compared to the TN_EP_STAS compartment, suggesting changes in tumor cell metabolism and energy utilization during dissemination (Supplementary Figure S3). Subsequently, we assessed the changes in DEGs in epithelial cells during carcinogenesis and dissemination, identifying 14 genes with a consistent increase in expression (Figure 4D; Supplementary Table S11). These genes are primarily involved and enriched in pathways such as the extracellular space/region, cellular iron ion homeostasis, carbohydrate metabolic process, and response to hydrogen peroxide (Figure 4E). To evaluate the clinical impact of these pro-cancer genes, we constructed a signature based on the expression of these 14 genes to predict LUAD patient prognosis and assessed it in the TCGA cohort (Figure 4F). Results showed that this signature has a significant correlation with both OS (*p* = 1.7e-05) and DFS (*p* = 0.035) in LUAD patients, thus highlighting its potential clinical relevance.

### Exploration of the tumor immune microenvironment (TIME) in TN_IM

**FIGURE 5:**
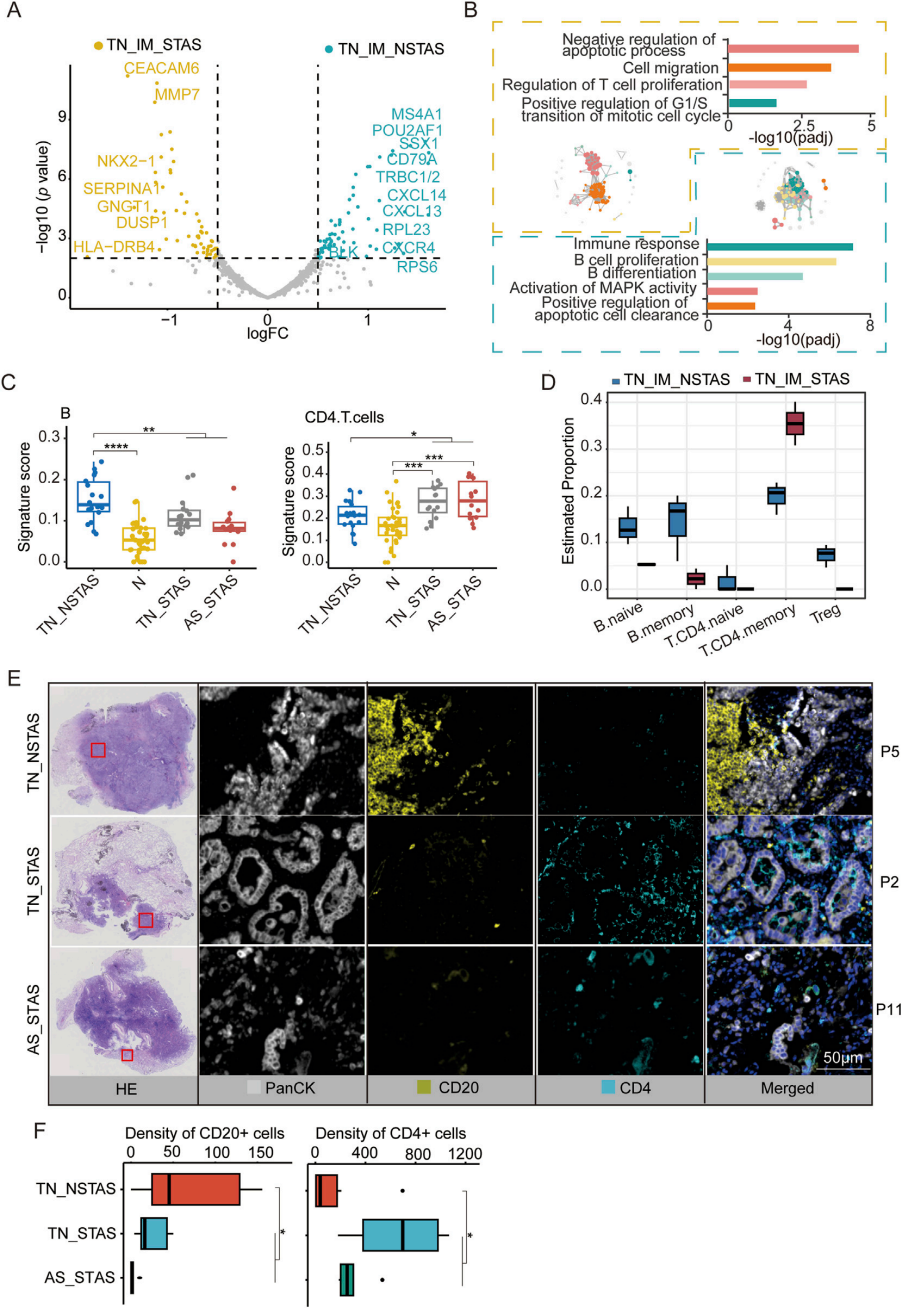
DEG analysis and cell abundance estimation in TIME between STAS and NSTAS groups. **(A)** DEGs between STAS and NSTAS in TN_IM. DEGs of TN_IM_STAS are highlighted in yellow, and DEGs of TN_IM_NSTAS are highlighted in cyan. **(B)** Functional pathway enrichment based on DEGs in TN_IM_STAS and TN_IM_NSTAS. TN_IM_STAS is shown in yellow, and TN_IM_NSTAS is shown in cyan. **(C)** Comparison of B and CD4^+^ T cells of TN_EP. **(D)** Comparison of B and CD4^+^ T cells from TN_IM. **(E)** Representative images from P5, P2, and P11 showing HE staining, PanCK (white), CD20 B cells (yellow), and CD4 T cells (cyan), as determined by mIF. Scale bar, 50 μm. **(F)** Density of the CD20- and CD4-positive cells among groups based on mIF data. DEGs, differentially expressed genes; TIME, tumor immune microenvironment; TN_IM_STAS, immune cell-enriched compartments within the tumor nest of STAS; TN_IM_NSTAS, immune cell-enriched compartments within the tumor nest of NSTAS. TN_NSTAS, tumor nest malignant epithelial cell-enriched compartments of NSTAS; TN_STAS, tumor nest malignant epithelial cell-enriched compartments of STAS; AS_STAS, airspace malignant epithelial cell-enriched compartments of STAS. *p < 0.05; **p < 0.01; ***p < 0.001; ****p < 0.0001.

Since the aberrant genes and pathways involved in STAS development suggest the involvement of immune and inflammatory responses in the AS spread, we focused our analysis on the TIME within the TN. *MS4A1, CD79A, POU2AF1, BLK,* and *CD37* were observed to be significantly highly expressed in the immune cell-enriched compartments of the NSTAS group (TN_IM_NSTAS), while genes such as *CEACAM6, MUC1*, *MMP7*, and *FBP1* were significantly highly expressed in the immune cell-enriched compartments of the STAS group (TN_IM_STAS) (Figure 5A; Supplementary Table S12). Further analysis revealed that some genes can significantly affect the prognosis of patients. Patients with a high expression of *BLK* have a significantly longer OS (*p* = 0.0043) and DFS (*p* = 0.015). Patients with a high expression of *LTB*, *CD37*, *MS4A1*, *CD79A*, and *POU2AF1* have a significantly longer OS (all *p* < 0.05) (Supplementary Figure S4). *BLK* contributes to immune cell pathways (such as B-cell proliferation and differentiation), indicating that the high expression of the *BLK* gene in the TN_IM_NSTAS group may regulate the TIME, and it has more immune cell enrichment and anti-tumor effects. As we expected, higher immune response and enriched B-cell proliferation were observed in the TIME of the TN_IM_NSTAS group, along with the enrichment of B-cell differentiation pathways and apoptotic cell clearance (Figure 5B).

The PanCK+ compartment can preliminarily explore the changes in the TIME (Carter et al., 2023). To explore deeper into the composition and proportion of various immune cells within the TIME, we evaluated immune cells in the tumor compartments of AOI using an immune cell signature score with the GSEA algorithm. The results indicated an increase in B cells within tumor regions compared to normal areas. However, with increased AS progression, the number of B cells significantly decreased. In contrast, CD4 T cells exhibited an opposite trend, with a noticeable increase in tumor regions and a significant increase in AS progression (Figure 5C; Supplementary Table S13).

These findings were confirmed by evaluation of the TIME in TN_IM with a deconvolution algorithm named SpatialDecon. Compared to TN_IM_STAS, TN_IM_NSTAS showed an increase in B cells, while CD4 T memory cells displayed the opposite trend (Figure 5D; Supplementary Table S14). Finally, mIF experiments were performed on NSTAS and STAS samples. The results further confirmed that the NSTAS group was characterized by a higher proportion of CD20-positive cells, whereas the STAS group showed a predominance of CD4-positive cells (Figures 5E,F; Supplementary Table S15). These suggest that the presence of B cells and CD4 T cells may play an important role in the progression of STAS.

### Differences in genes and pathways in the adjacent normal tissue between STAS and NSTAS

**FIGURE 6:**
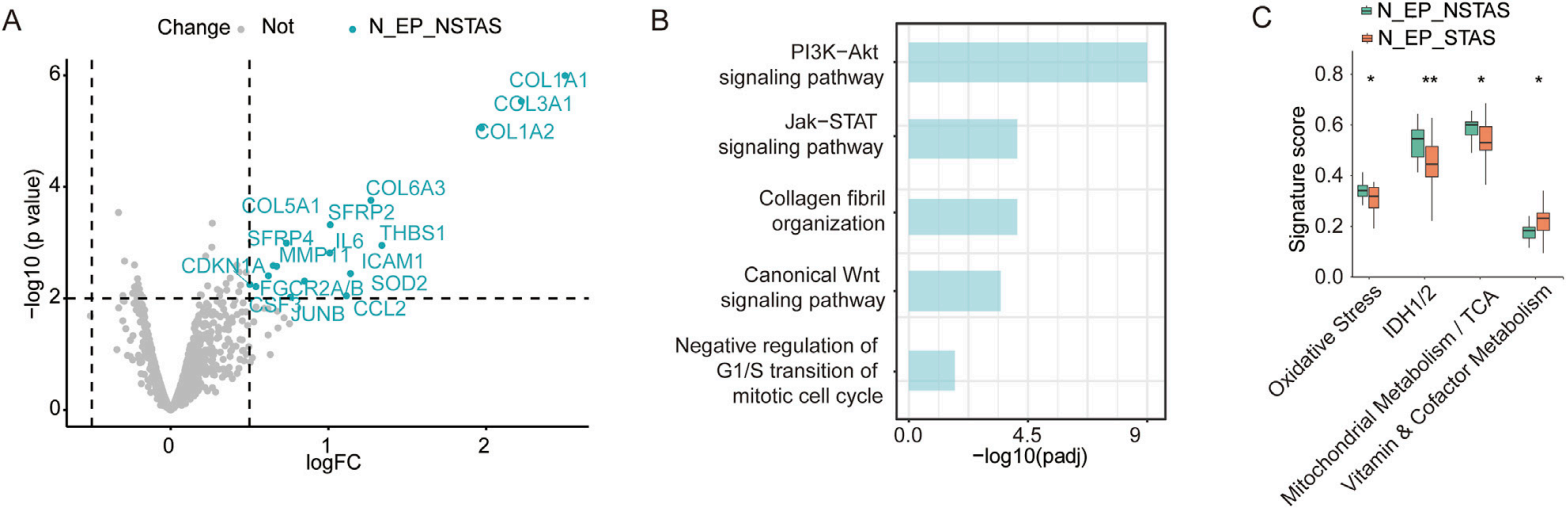
Comparison of adjacent normal tissue between N_EP_NSTAS and N_EP_STAS. **(A)** Volcano diagram representing DEGs between N_EP_STAS and N_EP_NSTAS. **(B)** Functional pathway enrichment of TN_EP_NSTAS. **(C)** Boxplot of the signature score between TN_EP_STAS and TN_EP_NSTAS. N_EP_STAS, epithelial cell-enriched compartments from adjacent normal tissue of STAS; N_EP_NSTAS, epithelial cell-enriched compartments from adjacent normal tissue of NSTAS. *p < 0.05; **p < 0.01.

To investigate whether there are differences in the underlying tissue components involved in AS during NSCLC development, we conducted a comparative analysis of adjacent normal epithelial cell-enriched compartments between STAS and NSTAS (N_EP_STAS and N_EP_NSTAS). The results revealed 17 genes, including *SFRP2*, *MMP11*, and *ICAM1*, that were significantly upregulated in the N_EP_NSTAS group. Additionally, COL family genes were notably suppressed in the N_EP_STAS group (Figure 6A). Pathway enrichment analysis of DEGs in N_EP_NSTAS indicated that epithelial cells in the NSTAS group exhibited more pronounced activation of traditional tumor-initiating pathways, such as PI3K-Akt, Wnt, and Jak-STAT. Meanwhile, the immune response pathways were also enhanced. In contrast, the G1/S transition of the mitotic cell cycle was significantly inhibited (Figure 6B), suggesting that, compared to NSTAS, STAS is more prone to accumulating somatic mutations at earlier stages and that the occurrence of STAS may involve mechanisms distinct from conventional tumor initiation pathways. Furthermore, signature analysis revealed that oxidative stress, IDH1/2, and TCA metabolism pathways were significantly more active in the NSTAS group, while vitamin metabolism was markedly enhanced in the STAS group (Figure 6C). This analysis indicated that NSCLC with and without AS development exhibit differences in gene dysregulation and pathways at the basal tissue level. In the STAS group, the lack of DNA cycle inhibition and immune response in the early stages of tumor initiation makes it more prone to accumulating cellular mutations, while also lacking effective mechanisms for clearing abnormal cells.

## Discussion

For patients with NSCLC, the occurrence of STAS significantly impacts the prognosis and influences the choice of surgical intervention, highlighting the importance of conducting comprehensive research on STAS. Traditional bulk-level molecular diagnostics struggle to accurately capture the heterogeneity of lung cancer tumors and the spatial regions of individual cell types (Song et al., 2023). Our study employed the GeoMx DSP spatial transcriptomics technology to segment multiple areas within samples based on cell type, thus allowing for the separate study of different cell types (Merritt et al., 2020). For the first time, this technology has been used to reveal the heterogeneity of tumor cells in NSCLC patients with and without STAS, as well as the molecular patterns of change in tumor cells within TNs and ASs. Additionally, based on the analysis of tumor epithelial cell regions, *HLA-DRB5* and *RASFRF1* are identified as promising molecular markers for indicating the occurrence of STAS and predicting patient prognosis.

The metabolism of tumors is flexible and varies according to the environment (Faubert et al., 2020). The Warburg effect is a preference for glycolysis and lactate secretion in the presence of oxygen, and it is a phenomenon of the metabolic characteristics of oncogenes under cellular autonomous control in many proliferating cancer cells and tumors (Vander Heiden et al., 2009). Our results show that the glycolytic pathway of lung cancer tumor cells is significantly higher than that of normal cells, which may also be a manifestation of the Warburg effect. Some studies have shown that glucose, fatty acids, amino acids, and glutamine metabolism have important impacts on the development of tumors (Du et al., 2022; Wu et al., 2023). For example, proliferative tumor cells have an increased demand for glutamine, and their catabolism, synthetic metabolism, and transportation are crucial for the survival and development of tumor cells (Du et al., 2022). Differences in pathways related to DNA damage repair, cell cycle arrest, and apoptosis evasion further characterize lung cancer tumor cells and contribute to their drug resistance (Wu et al., 2023; Huang and Zhou, 2021). Therefore, our results are consistent with the trend of the reported scientific research, indicating that tumor cells exhibit more metabolic abnormalities and cellular dysfunction, which may contribute to the occurrence of lung adenocarcinoma.

When we compared PanCK+ tumor areas between STAS (containing TN_EP and AS_EP) and NSTAS (containing TN_EP) patients, the result revealed a significant decline in the expression of the *HLA-DRB5* gene in the STAS group. Recent studies have shown that high-risk lung cancer groups have significantly lower *HLA-DRB5* expression than normal samples and low-risk groups, with *HLA-DRB5* being significantly lower in malignant epithelial cells than in normal ones (Wang L. et al., 2022; Qin et al., 2024; Xu et al., 2022). Analysis from the TCGA database further demonstrates that NSCLC patients with low expression of *HLA-DRB5* have significantly prolonged survival. The HLA-DRB family genes are notably lesser in number in NSTAS patients than in NSCLC patients, , suggesting an increase in tumor immune evasion, reduction of T-cell recognition and activation, and a further confirmation of decreased immunogenicity of cancer cells (Wang L. et al., 2022; Datar et al., 2021). In colorectal cancer, *HLA-DRB5* expression levels are significantly correlated with the infiltration of CD8^+^ T cells, CD4^+^ T cells, and dendritic cells (Morafraile et al., 2023), implying that tumors in the NSTAS group of our samples might exhibit greater immune cell infiltration. The DEGs pathways enriched in TN_NSTAS also reflect an upregulation in inflammatory response pathways. Additionally, compared with TN_NSTAS, we found *RASGRF1* significantly downregulated in TN_STAS, which also markedly impacts LUAD prognosis; *RASGRF1* was primarily enriched in immune-related pathways such as the “B cell receptor signaling pathway” and the “chemokine signaling pathway,” and *RASGRF1* was significantly positively correlated with the infiltration levels of immune cells such as B+ cells, CD8^+^ T cells, CD4^+^ T cells, macrophages, and neutrophils (Li et al., 2021). This study is the first to propose that the *HLA-DRB5* gene in NSCLC tumor cells may inhibit the occurrence of STAS, and NSTAS samples showing high *HLA-DRB5* expression potentially indicate higher immune cell infiltration and an anti-immune response.

It is interesting to note that in our study, we found significant differential expression of genes between AS and TNs in the STAS group, including *CXCL14* and *THBS1*, which showed significantly higher expression in the AS. *CXCL14*, also known as the chemokine expressed in the breast and kidney, is a member of the C–X–C motif chemokine ligand family. In NSCLC, *CXCL14* promotes tumor metastasis through *ACKR2*. Consistent with expectations, the EMT signature score was significantly higher in the AS_EP_STAS compartment than in the TN_EP_STAS compartment, highlighting the crucial role of the EMT pathway during dissemination. The involvement of *ACKR2* in CXCL14-triggered signaling pathways, such as phospholipase Cβ3, protein kinase Cα, and proto-oncogene tyrosine-protein kinase Src, which subsequently upregulate nuclear factor kappa B transcriptional activity, leads to cancer cell EMT and migration (Chang et al., 2023). Additionally, the enrichment of the TGF-β signaling pathway in AS_STAS may suggest that *CXCL14*-induced fibroblast production of TGF-β enhances lung cancer invasion and migration (Xu et al., 2024). Pathologically, overexpression of TGF-β leads to EMT, extracellular matrix deposition, and cancer-associated fibroblast formation, contributing to fibrotic diseases and cancer (Peng et al., 2022).

*THBS1* is an adhesive glycoprotein mediating cell–cell and cell–matrix interactions, regulating autophagy, senescence, and stem cell maintenance (Kaur et al., 2021). In NSCLC patients, high *THBS1* expression is associated with poorer prognosis and resistance to osimertinib treatment (Li et al., 2022; Kosibaty et al., 2022). Furthermore, *THBS1* is a core regulatory target of the TGF-β pathway, and a positive feedback loop between *THBS1* and the TGF-β pathway is a primary reason for drug resistance (Lee et al., 2023). Our research findings suggest that TGF-β-mediated EMT may be the cause of AS dissemination in NSCLC. For the first time in our study, the spatial transcriptome technology was applied to elucidate the differential gene expression of tumor cells between TN and ASs, which will have an important driving role in the study of the mechanism of STAS and clinical treatment.

Our study, utilizing spatial transcriptomics technology for the first time, clarified the molecular characteristics and pathway differences between TN_STAS and AS_STAS tumor cells in NSCLC, thus significantly contributing to the understanding of AS mechanisms and clinical treatment. By analyzing DEGs from tumor initiation to spread and categorizing them into patterns, we identified 14 genes that are continuously overexpressed during cancer initiation and spread as Pro-AS genes. Based on the expression levels of this Pro-AS signature, prognosis prediction in TCGA LUAD patients showed significant stratification (both *p* < 0.05), marking a significant advance in the study of AS mechanisms and their clinical implications.

Additionally, we conducted a preliminary exploration at the microenvironment level and identified differences in CD45^+^ immune cell compartments between the STAS and NSTAS groups. *MS4A1* and *BLK* were significantly overexpressed in TN_IM_NSTAS. The gene *BLK*, a non-receptor tyrosine kinase of the Src family located on chromosome 8p3.1, primarily regulates the proliferation and differentiation of B lymphocytes (Xu et al., 2023). Moreover, *BLK* modulates the immune tolerance in B lymphocytes. Overexpression of *BLK* can inhibit NSCLC growth by activating the apoptosis pathway, inhibiting the G2M checkpoint, and suppressing the glycolysis pathway (Xu et al., 2023). *MS4A1*, the gene encoding the B-cell surface marker CD20, is significantly downregulated in human colorectal cancer. Additionally, the expression level of *MS4A1* in colorectal cancer correlates positively with patient survival rates (Mudd et al., 2021). *CD37* is selectively expressed on mature B cells, with limited or no expression on other hematopoietic cells such as T cells and NK cells, granulocytes, monocytes, and dendritic cells. *CD37* has signal transduction ability because it contains functional ITIM-like and ITAM-like motifs within cells, which play a role in promoting the survival and apoptosis signal transduction through the PI3K/AKT pathway (Oostindie et al., 2020).

Subsequent deconvolution analysis was used to assess the composition and proportion of immune cells, revealing significant infiltration of B cells in the TN_EP_NSTAS compartment, while CD4 T cells were more inclined to infiltrate TN_EP_STAS. This result was also validated in the TN_IM areas of our cohort. B-cell infiltration is associated with the therapeutic efficacy and prognosis of NSCLC and is linked to the presence of tertiary lymphoid structures (Patil et al., 2022; Federico et al., 2022), suggesting that NSTAS patients may have a better prognosis. Our transcriptomic data from immune regions suggest that compared to STAS, NSTAS exhibits a “hotter” tumor microenvironment within TN, which is likely reflected in the enrichment and activity of B cells.

Despite these important discoveries, we acknowledge certain potential limitations of our study. First, the DSP analysis focused on selecting PanCK+ epithelial cell compartments, with fewer selections made in the CD45^+^ immune-enriched compartments, thus posing limitations to our study of the TIME; in future research, we aim to further investigate the STAS microenvironment. Second, the number of panel genes detected in spatial transcriptomics is relatively small, so it is necessary to conduct spatial whole-transcriptome detection in a later stage to further explore the information. Third, not all patients have obtained OS and PFS data, and we cannot clearly analyze the correlation between *HLA-DRB5/RASGRF1* expression in the TN with PFS or OS. Therefore, in future research, we plan to explore this subset of patients and collect additional PFS and OS data to enhance our findings.

## Conclusion

Our study marked the first application of the spatial transcriptomics technology to investigate STAS in NSCLC, suggesting the potential of using *HLA-DRB5* expression in the spatial region as a spatial marker for inhibiting STAS, and enriched the research on molecular markers for STAS. Using the advantages of DSP spatial transcriptomics, for the first time, we confirmed the molecular heterogeneity between TN and AS in the tumor compartment. The findings indicated that TGF-β-mediated EMT may be a key mechanism for the occurrence of air space spread in NSCLC. These insights aimed to advance the scientific understanding of STAS and move toward the precise diagnosis and treatment of STAS.

## Data Availability

The data presented in the study are deposited in the GSA-Human repository, accession number HRA012209.

## Supplementary material

SUPPLEMENTARY FIGURE S1 Survival curves of OS and DFS in LUAD of HLA-DRB5 expression.

SUPPLEMENTARY FIGURE S2 Expression levels of differentially expressed genes between TN_STAS and AS_STAS across different groups.

SUPPLEMENTARY FIGURE S3 Significant difference signatures between TN_EP_STAS and AS_EP_STAS.

SUPPLEMENTARY FIGURE S4 Survival curves of OS and DFS in LUAD of different gene expression.

SUPPLEMENTARY TABLE S1 Clinical characteristics of patients.

SUPPLEMENTARY TABLE S2 Differential DNA expression between tumor and normal groups.

SUPPLEMENTARY TABLE S3 Cell function signature score estimation in tumor and normal groups.

SUPPLEMENTARY TABLE S4 Metabolism signature score estimation in tumor and normal groups.

SUPPLEMENTARY TABLE S5 Differential RNA expression between STAS and NSTAS in tumors.

SUPPLEMENTARY TABLE S6 Cell density of Panck- and HLA_DRB5-positive cells estimated by multiplex immunofluorescence.

SUPPLEMENTARY TABLE S7 Metabolism signature score estimation in T_STAS and T_NSTAS groups.

SUPPLEMENTARY TABLE S8 Metabolism signature score estimation in TN_STAS and TN_NSTAS groups.

SUPPLEMENTARY TABLE S9 Cell density of Panck-, NT5E-, and ITGA2-positive cells estimated by multiplex immunofluorescence.

SUPPLEMENTARY TABLE S10 Cell function signature score estimation in TN_STAS and AS_STAS groups.

SUPPLEMENTARY TABLE S11 Pro-AS genes used to construct the prognostic prediction signature.

SUPPLEMENTARY TABLE S12 Differential RNA expression between IM_STAS and IM_NSTAS groups.

SUPPLEMENTARY TABLE S13 Immune cell signature score estimated by GSEA.

SUPPLEMENTARY TABLE S14 Cell type proportion estimated by Spatialdecon in IM.

SUPPLEMENTARY TABLE S15 Cell density of CD4- and CD20-positive cells estimated by multiplex immunofluorescence.

## Abbreviations

AOI, area of illumination or area of interest; AS, airspace; AS_EP, airspace malignant epithelial cell-enriched compartments; CTA, cancer transcriptome atlas; DEGs, differentially expressed genes; DSP, digital spatial profiling; FFPE, formalin-fixed paraffin-embedded; HE, hematoxylin and eosin; ITH, intra-tumor heterogeneity; KM, Kaplan–Meier; mIF, multiplex immunofluorescence; N, normal tissues; N_EP, normal epithelial cell-enriched compartments; NSCLC, non-small-cell lung cancer; OS, overall survival; PCA, principal component analysis; RFS, recurrence-free survival; ROI, regions of interest; STAS, spread through air spaces; TFC, template free control; TN, tumor nests; TN_EP, tumor nest malignant epithelial cell-enriched compartments; TN_IM, immune cell-enriched compartments; UMAP, uniform manifold approximation and projection.
