# Supplementary figures and images for: Spatial deciphering of the transcriptomic heterogeneity of tumor spread through air spaces in lung cancer
Source: Front Pharmacol. 2025 Jul 25;16:1567527. doi: 10.3389/fphar.2025.1567527 (PMC12331751; doi:10.3389/fphar.2025.1567527)

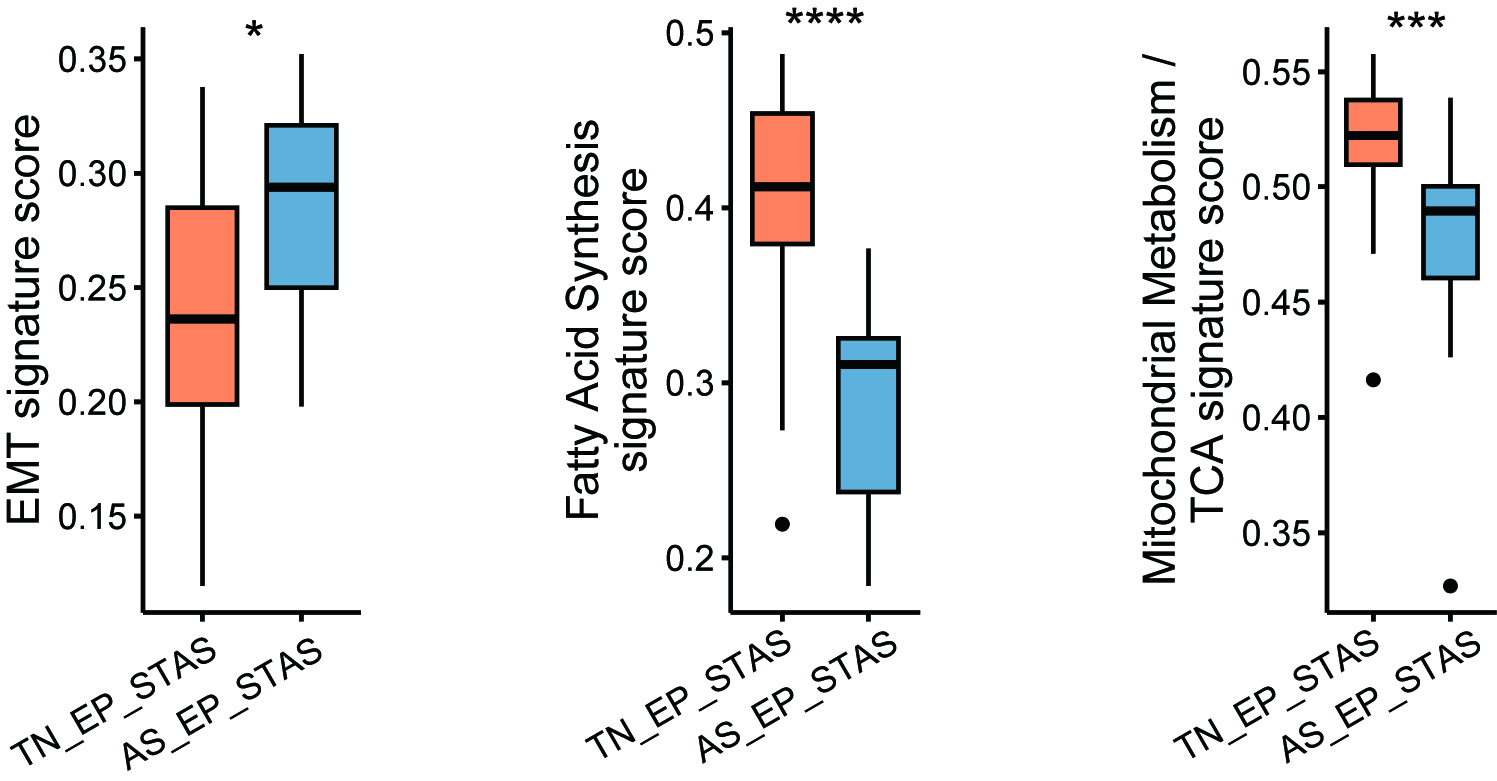

Supplement: Supplementary file 1 [file Image3.tif]

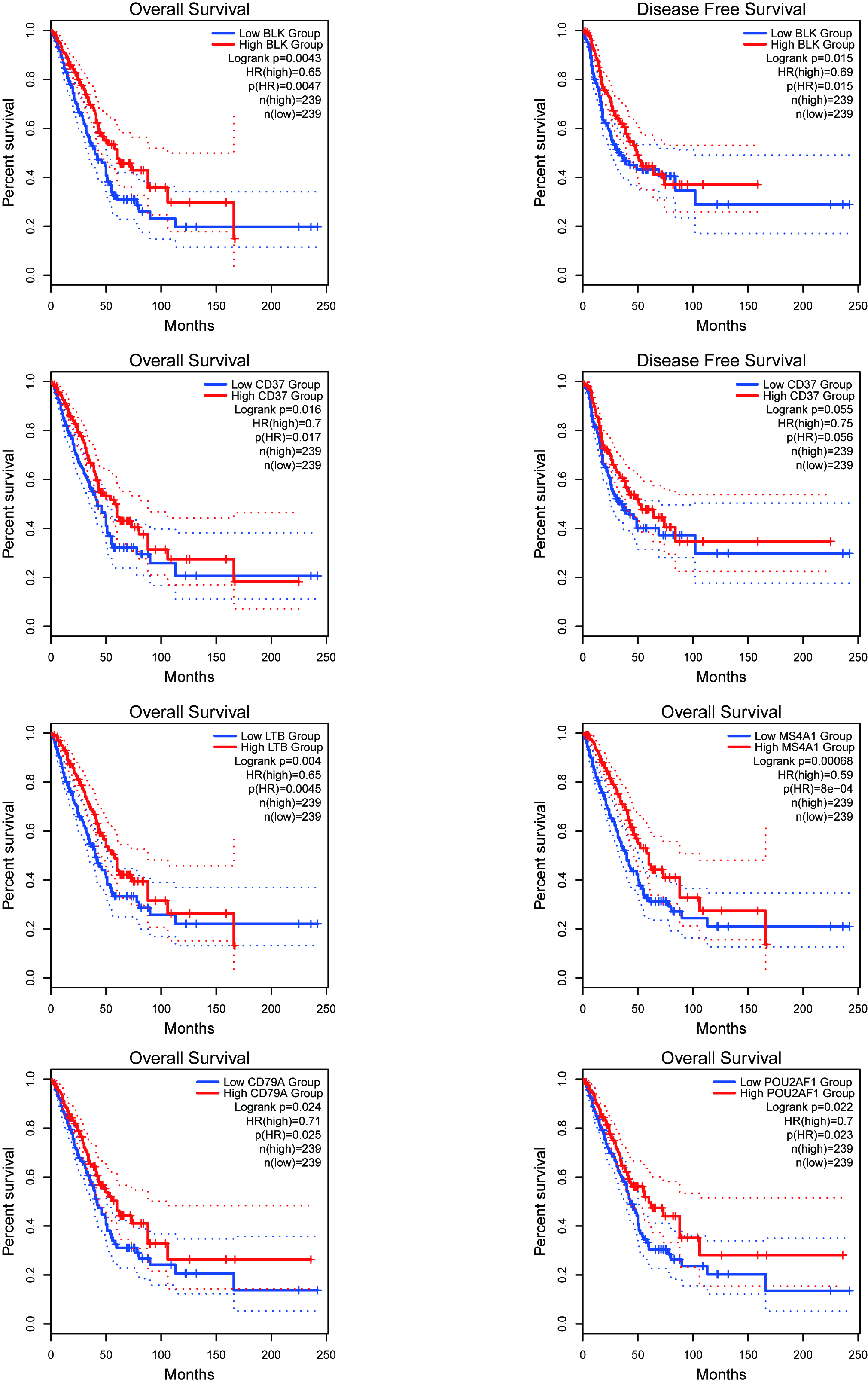

Supplement: Supplementary file 2 [file Image4.tif]

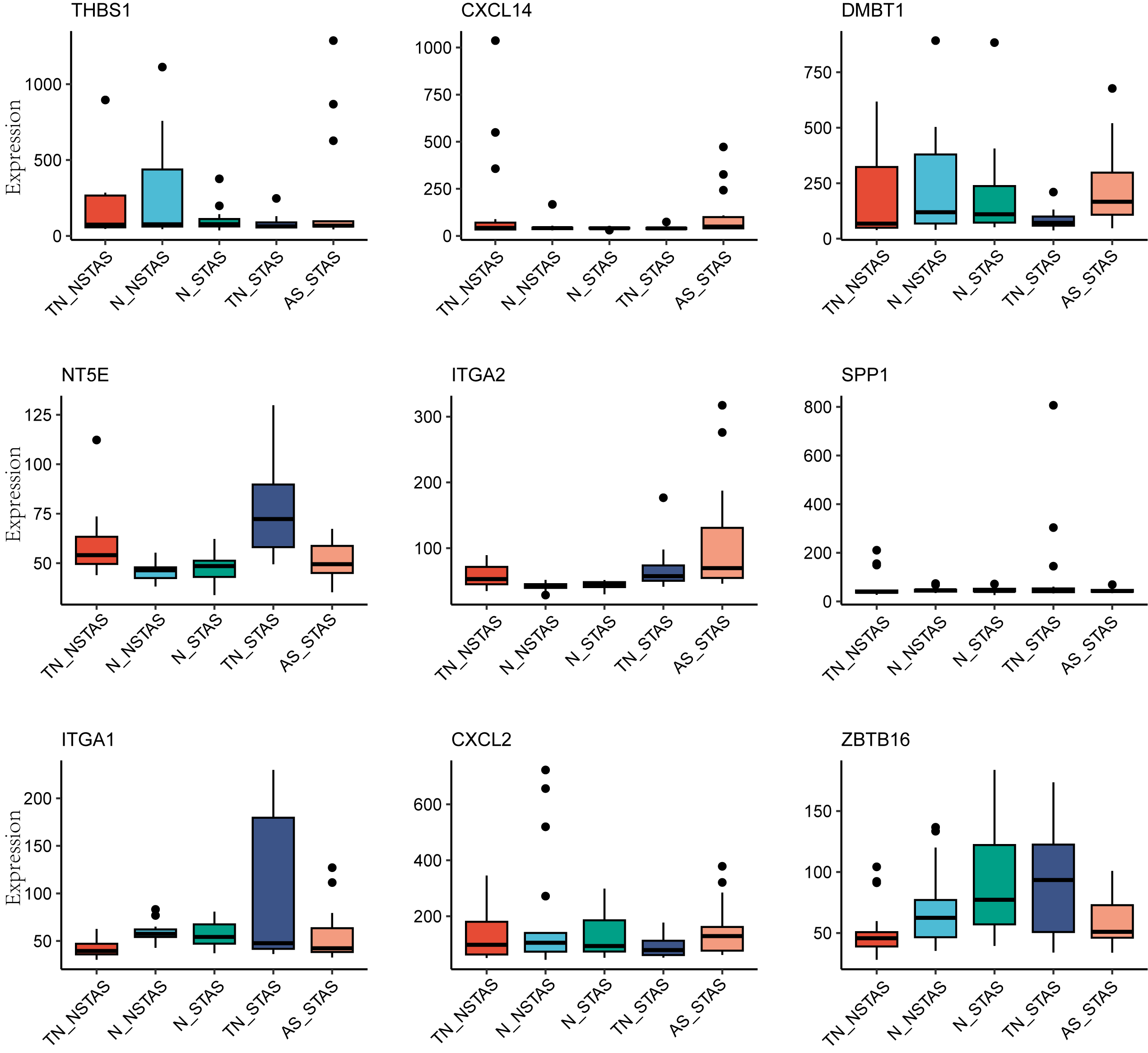

Supplement: Supplementary file 4 [file Image2.tif]

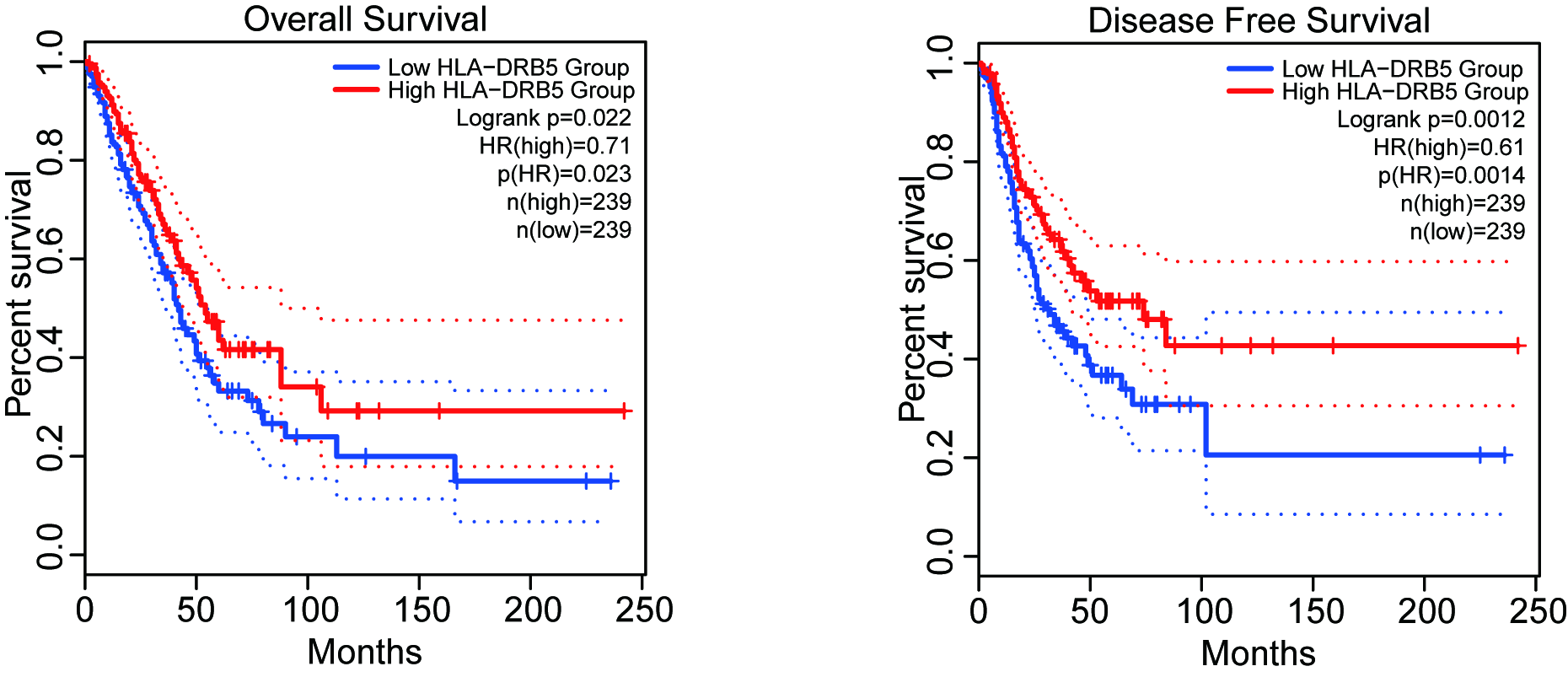

Supplement: Supplementary file 5 [file Image1.tif]
